# Hydroxyapatite-Coated Ti6Al4V ELI Alloy: In Vitro Cell Adhesion

**DOI:** 10.3390/nano14141181

**Published:** 2024-07-11

**Authors:** Marco Ruggeri, Dalila Miele, Laura Caliogna, Eleonora Bianchi, Johannes Maui Jepsen, Barbara Vigani, Silvia Rossi, Giuseppina Sandri

**Affiliations:** 1 Department of Drug Sciences, University of Pavia, Viale Taramelli 12, 27100 Pavia, Italy; marco.ruggeri@unipv.it (M.R.); dalila.miele@unipv.it (D.M.); eleonora.bianchi01@unipv.it (E.B.); barbara.vigani@unipv.it (B.V.); silvia.rossi@unipv.it (S.R.); 2 Orthopaedic and Traumatology, Fondazione IRCCS Policlinico San Matteo, 27100 Pavia, Italy; l.caliogna@smatteo.pv.it; 3 Stryker Trauma GmbH, Professor Küntscher-Straße 1-5, 24232 Schönkirchen, Germany; maui.jepsen@yahoo.de

**Keywords:** titanium alloy, Ti6Al4V ELI, hydroxyapatite, osteodifferentiation, osteointegration

## Abstract

The high rate of rejection and failure of orthopedic implants is primarily attributed to incomplete osseointegration and stress at the implant-to-bone interface due to significant differences in the mechanical properties of the implant and the surrounding bone. Various surface treatments have been developed to enhance the osteoconductive properties of implants. The aim of this work was the in vitro characterization of titanium alloy modified with a nanocrystalline hydroxyapatite surface layer in relative comparison to unmodified controls. This investigation focused on the behavior of the surface treatment in relation to the physiological environment. Moreover, the osteogenic response of human osteoblasts and adipose stem cells was assessed. Qualitative characterization of cellular interaction was performed via confocal laser scanning microscopy focusing on the cell nuclei and cytoskeletons. Filipodia were assessed using scanning electron microscopy. The results highlight that the HA treatment promotes protein adhesion as well as gene expression of osteoblasts and stem cells, which is relevant for the inorganic and organic components of the extracellular matrix and bone. In particular, cells grown onto HA-modified titanium alloy are able to promote ECM production, leading to a high expression of collagen I and non-collagenous proteins, which are crucial for regulating mineral matrix formation. Moreover, they present an impressive amount of filipodia having long extensions all over the test surface. These findings suggest that the HA surface treatment under investigation effectively enhances the osteoconductive properties of Ti6Al4V ELI.

## 1. Introduction

The high rate of rejection and failure in orthopedic implants is primarily attributed to incomplete osseointegration and stress at the implant–bone interface due to significant differences in the mechanical properties of the implant and the surrounding bone [1,2].

Osseointegration, consisting of early migration and proliferation of osteoblasts and the subsequent synthesis, deposition, and mineralization of the bone matrix, is the result of structural and functional integration between the living bone tissue and the surface of the implant [3,4]. The rate and success of osseointegration are heavily influenced by the topography and chemical composition of implant surfaces [5]. These properties play a crucial role in the host’s biological response, guiding cells to proliferate and differentiate, as well as to produce and release growth factors stimulating healing [6]. During the initial phases of implant integration, for example, a certain degree of surface roughness is desirable as it has been associated with improved cellular adhesion, proliferation, differentiation, synthesis of the extracellular matrix components, as well as the production of alkaline phosphatase, osteocalcin, TGF-β, and PGE2 [7,8]. The activation of specific signals influences the cells’ conformation and guides them toward osseous differentiation. This occurs thanks to focal adhesions, protein complexes that bind the cytoskeleton to the extracellular matrix. Among these, vinculin plays a crucial role as it is part of adherent junctions and supports to anchor cells to cells and cells to the extracellular matrix, while osteoprotegerin and the bone resorption inhibitor glycoprotein reduce osteoclastic activity [9,10].

Due to its mechanical properties, corrosion resistance, and biocompatibility, titanium (Ti) and its alloys are widely used for orthopedic implants [11]. Ti6Al4V, featuring 6% aluminum and 4% vanadium, in particular, has become prominent in the biomedical field, and it is characterized by a higher modulus of elasticity than pure titanium [12].

To enhance the implants’ bone–tissue interaction, various surface treatments have been developed for orthopedic implants. Recent advancements in titanium implant technology have focused on enhancing osseointegration, reducing infection risks, and improving long-term success rates. Latest studies have explored innovative surface modifications, alloy compositions, and bioactive coatings to achieve these goals. In particular, a promising approach in this field is the hydroxyapatite (HA) coating on titanium implants [13,14]. These have been proven to add an improved osteogenic response, accelerating osteoblast adhesion and bone healing [15]. HA is a naturally occurring mineral form of calcium apatite that is present in bones and teeth, and in particular, bones are made primarily of HA crystals (70%) interspersed in a collagen matrix. For these reasons, HA confers increased hydrophilicity of titanium implants, improving osteoconductivity and osseointegration and accelerating bone healing. Moreover, as it is chemically similar to the mineralized phase of the bone, it is considered highly biocompatible [16].

Given these premises, the aim of this work was the in vitro characterization of Ti6Al4V ELI alloy featuring a novel surface treatment of nanocrystalline hydroxyapatite with super hydrophilic properties. This investigation focuses on the in vitro behavior of the material following exposure to the physiological environment. Moreover, the interaction and osteogenic response of human osteoblasts and adipose stem cells to the material was assessed. Finally, qualitative imaging of the cellular structure focusing on nuclei, cytoskeletons, and filipodia was conducted via confocal laser scanning microscopy as well as scanning electron microscopy. In this work, the performance comparison among raw Ti6Al4V ELI, anodized Ti6Al4V ELI, and HA surface-modified Ti6Al4V ELI has been investigated using a multidisciplinary approach to characterize the properties not only from preclinical in vitro (cell adhesion and extracellular matrix production) but also from the physicochemical point of view (Ca^2+^ release and extracellular protein adhesion).

## 2. Materials and Methods

### 2.1. Materials

Ti6Al4V ELI coupons with anodization type II surface treatment modified with nanocrystalline HA surface layer, according to [17,18] (D 9 × 2 mm) (HA modified), served as test samples, while Ti6Al4V ELI coupons with anodization type II surface treatment (D 9 × 2 mm) (AnoII) were used as controls. The sample size and selection criteria were determined based on the feasibility of in vitro testing, aiming to detect significant preclinical differences with high confidence. All samples were cleaned and sterilized by gamma irradiation prior to evaluation.

### 2.2. Methods

#### 2.2.1. Coating Morphology and Degradation

The coupons were dipped in 1 mL PBS (phosphate-buffered saline, 6.6–7.2 pH; 137 mM NaCl, 2.7 mM KCl, 9.5 mM phosphate buffer, without magnesium or calcium—VWR, Biosigma, Italy) and at different time points (0, 4, 7, 14, 21 days) were imaged with scanning electron microscope (Mira3XMU, Tescan, Czech Republic, CisRic, Arvedi, University of Pavia) without sputtering, at 8 kV voltage, high vacuum, and room temperature, to assess changes in the surface morphology.

Moreover, an energy-dispersive X-ray analyzer (EDX, Mira3XMU, Tescan, Czech Republic, CisRic, Arvedi, University of Pavia) was used to determine the hydroxyapatite coating.

#### 2.2.2. Protein Adhesion

Both HA-modified and AnoII coupons were placed in 0.5 mL of a 10 μg/mL *w*/*w* FITC–albumin or FITC–fibronectin solution in PBS (pH 7.2) and incubated at 37 °C in a shaking bath. At 15, 30, 120, and 240 min, the supernatants (100 µL) were collected from the wells, and fluorometric assays were performed using a FLUOstar^®^ Omega Microplate Reader (BMG LABTECH, Milan, Italy) in order to quantify the protein amount remaining in the solution [2]. After 240 min, the coupons were washed with 0.5 mL PBS to remove the loosely bound proteins, and further measurements were performed on the washing media. Next, 100 µL of each solution was placed in a 96 well-plate, and the fluorescence was recorded by means of a spectrofluorometer at an excitation wavelength λex = 500 nm and emission wavelength λem = 520 nm. The amount of proteins adhered to the sample surface was calculated as the difference between the initial amount of protein in the medium and the protein amount that remained in the PBS solution after contact with the coupons.

Furthermore, confocal laser scanning microscopy (CLSM) analysis was performed in order to visualize the protein adhered to both surfaces after 240 min. Following the removal of loosely bound proteins as previously described, the coupons were placed on glass slides and analyzed using CLSM (Leica TCS SP2, Leica Microsystems, Buccinasco, Italy) at λex = 501 nm and λem = 523 nm for FITC–albumin or FITC–fibronectin. A 3D reconstruction of the protein layer adhered to the surfaces was performed.

#### 2.2.3. Adhesion, Proliferation, and Differentiation of Human Osteoblasts and Adipose-Derived Stem Cells

Human osteoblasts (HOBs, C-12720, Promocell, Milan, Italy) were cultured in a T-75 tissue culture flask with Osteoblast Growth Medium (Cell Applications Inc., Merck, Rome, Italy), supplemented with 10% *v*/*v* fetal bovine serum (FBS, Biowest, Bradenton, FL, USA) and with 200 IU/mL penicillin/0.2 mg/mL streptomycin (Lonza, Durham, NC, USA), kept at 37 °C in a 5% CO_2_ atmosphere with 95% relative humidity (RH).

Human adipose-derived stem cells (hASCs, ZenBio, Durham, NC, USA) were grown in basal-α-MEM medium based on 1% of minimum essential medium (MEM, ZenBio, Durham, NS, USA), supplemented with 10% FBS, 0.22% *w*/*v* sodium bicarbonate, and with penicillin/streptomycin solution (Lonza, Pero, Italy) at 37 °C in a 5% CO_2_ atmosphere.

##### Cell Proliferation and Differentiation

**Figure 1:**
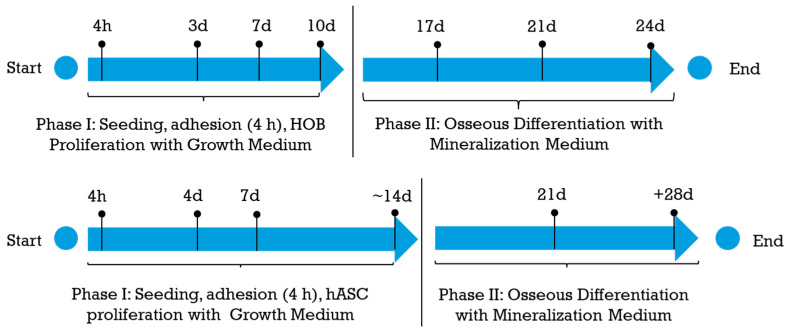
Schematic of cell experiments.

Figure 1 shows a schematic of cell experiments.

Both HA-modified and AnoII coupons were placed at the well bottom in a 48-well plate. Cells were seeded onto each coupon at 40 × 10^3^ cells/well seeding density in the case of HOBs and 30 × 10^3^ cells/well in the case of hASCs and allowed to attach. The difference in seeding density has been the consequence of the greater dimensions of hASCs compared to HOBs. After 4 h, each coupon was moved to a new well, and fresh growth medium was added. Subsequently, HOB and hASC proliferation were monitored. After 10 or 14 days of proliferation (phase I), the Osteoblast Growth Medium was changed to Osteoblast Mineralization Medium (Basal Medium plus Supplement Mix, Promocell, Italy) (phase II) to promote the differentiation of both HOBs and hASCs up to 24 or 28 days. Fresh growth medium was provided every 3 days. At predefined time points, a 10% *v*/*v* AlamarBlue™ (ThermoFisher Scientific, Waltham, MA, USA) solution was prepared in the respective growth medium, and 400 μL of the AlamaBlue™ solution was dispensed in each well and incubated at 37 °C for 3 h. Afterward, the reduced alamarblue™ was transferred in a 96-well plate, and the fluorescence was read using λex 560 nm and λem 590 nm.

##### Cell Imaging

HOB or hASC morphology and distribution onto the coupons were imaged via CLSM at different time points. The coupons were dipped in 4% *v*/*v* paraformaldehyde (PFA, Sigma Aldrich, Rome, Italy) solution for 20 min at room temperature and washed three times in PBS for 20 min to fix the cells. Subsequently, they were dipped in PBS containing 0.1% *v*/*v* Triton X-100 (Bio-Rad Laboratories, Hercules, CA, USA) for 5 min to obtain cell permeabilization; then, they were washed three times with PBS for 5 min [19,20]. As for hASCs, the extracellular matrix was stained using anti-collagen I rabbit polyclonal antibody (Thermofisher, Monza, Italy; 100 µL/sample at 10 µg/mL in PBS) to immuno-labeled collagen I [21]. After cell fixation, cell cytoskeletons were fixed with Phalloidin Atto 488 (FITC) (10 nM diluted 1:40, Sigma, Italy) for 40 min to stain cellular cytoskeletons and then washed 3 times with PBS for 5 min each in the dark, then cell nuclei were stained with 0.1 µg/mL Hoechst 33258 (Sigma, Italy) and were washed twice with PBS for 5 min. Cell substrates were placed onto a microscope slide and imaged immediately using a CLSM (Leica TCS SP5/8, Leica Microsystems, Buccinasco, Italy). For both cell types, cytoskeletons and nuclei were visualized at the following wavelengths: phalloidin Atto 488: λex = 495 nm and λem = 520 nm; Hoechst 33258: λex = 346 nm − λem = 460 nm, while collagen I (ATTO 488 goat anti-rabbit IgG) was at λex = 501 nm and λem = 523 nm.

After CLSM analysis, the coupons were sputtered with 5 nm of gold (Luxor-Tech Gmbh, Langen, Germany) and imaged with SEM (Phenom Pro, Thermo-Scientific, Alfatest, Italy) at 9–15 kV voltage, high vacuum, and at room temperature.

##### Osteogenic Characterization

The osteogenic response was investigated by Rt-qPCR. The well bottom (CTRL) was considered as a reference substrate. The qPCR (quantitative polymerase chain reaction) was performed after 7 and 14 days of differentiation after initial proliferation. Five different genes associated with the osteogenic response were considered: alkaline phosphatase (ALPL), runt-related transcription factor 2 (RUNX2), bone gamma-carboxyglutamate (gla) protein (BGLAP) encoding for osteocalcin, collagen I, and secreted phosphoprotein 1 (SPP1) encoding for osteopontin. The glyceraldehyde 3-phosphate dehydrogenase (GADPH) expression was used for normalization of the qPCR data, and the ΔΔCt method was applied.

For each of the respective time points, the total RNA was isolated with a TriZol agent (Merck, Italy) according to the manufacturer’s instructions and quantified spectrophotometrically at 230 nm by means of a FLUOstar Omega Microplate Reader equipped with a L-Vis microplate. Next, 1 µg of RNA was employed as a template for the synthesis of the cDNA. Reverse transcription was carried out using the SimpliAmp™ Thermal Cycler (Applied Biosystems™ LS4376357, Thermo-Fisher Scientific, Milan, Italy) and following the manufacturer’s instructions of the iScript™ cDNA Synthesis Kit (BioRad, Segrate, Italy). The expression of the ALPL, RUNX2, BGLAP, COL1A, and SPP1 coding RNAs was analyzed by quantitative rt-PCR using Sso Advanced Universal SYBR Green Supermix (Biorad, Segrate, Italy), and each specific primer was set at a final concentration of 400 nM for 50 ng of cDNA. The sequences and the amplicon length of the primers involved in the study are listed in Table S1.

The thermal cycling program was performed by means of a StepOnePlus™ Real-Time PCR System (Thermo Fisher Scientific, Waltham, MA, USA), set as follows: polymerase activation was achieved in 30 s at 95 °C; DNA denaturation at 95 °C for 15 s, and annealing at 60 °C for 30 s repeating cycles 40 times. Finally, melt curves were recorded.

## 3. Results and Discussion

### 3.1. SEM Analysis

**Figure 2:**
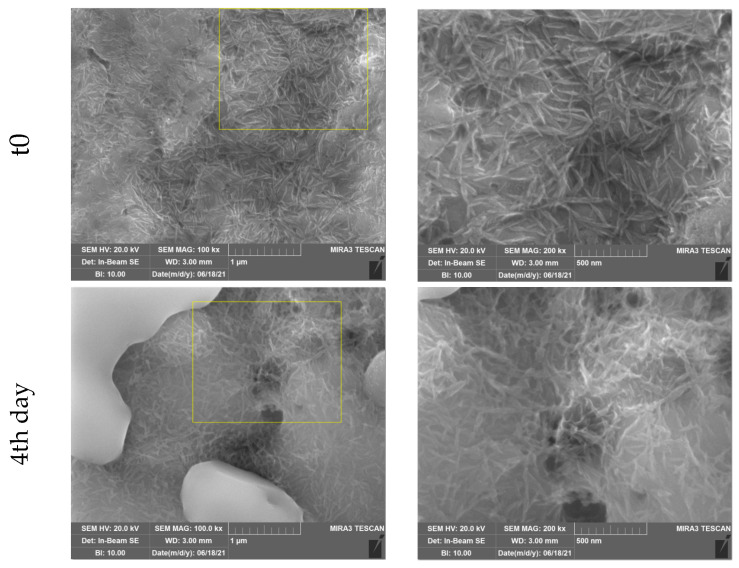

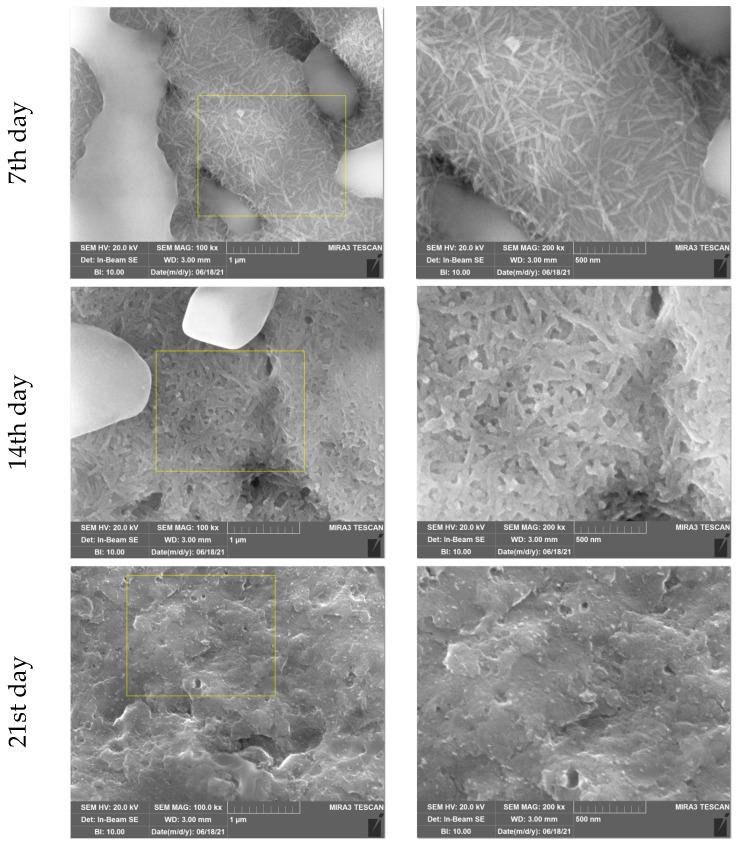
SEM micrographs of HA-modified samples at time 0 and after 4, 7, 14, and 21 days of dissolution at 100 k and 200 k magnification (yellow squares magnified).

Figure 2 shows the SEM images of HA-modified coupons after 0, 4, 7, 14, and 21 days of exposure to PBS at two different magnifications.

The HA-modified coupons are coated with an ultra-thin layer of nanocrystalline HA featuring a distinct needle-shaped pattern. The EDX spectra showed the presence of P and Ca in HA-modified samples, confirming the hydroxyapatite coating (Figure S1). Following exposure to PBS for up to 7 days, there were no major changes to the morphological appearance of the surface. Yet, after 14 days of exposure, the HA surface treatment changed its appearance to a more rod-shaped structure. Following the 21st day of exposure, the distinct needle-like structures were no longer visible. The change in morphology and the subsequent disappearance of the nanoscopic structure could be attributed to the solubilization of the hydroxyapatite layer, a common behavior associated with HA coatings [22].

### 3.2. Protein Adhesion

**Figure 3:**
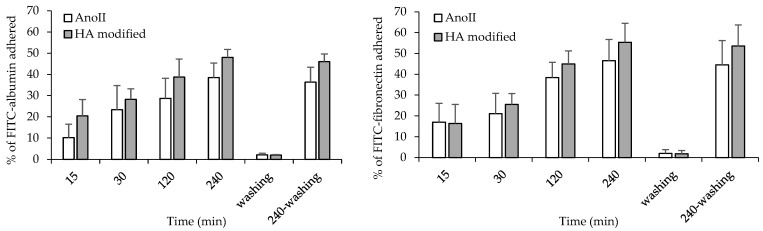
Percentage of FITC–albumin (**left panel**) and FITC–fibronectin (**right panel**) adhered onto Ti6Al4V ELI coupons with anodization type II surface treatment (AnoII) and Ti6Al4V ELI coupons with anodization type II surface treatment—HA modified (mean values ± s.d.; n = 3). Mann–Whitney test (Statgraphics Centurion)—*p* < 0.01 = top panel—AnoII vs. HA modified 120 min, AnoII vs. HA modified 240 min, AnoII vs. HA modified 240 min—washing; bottom panel—AnoII vs. HA modified 240 min, AnoII vs. HA modified 240 min—washing.

Figure 3 shows the results of the FITC–albumin (top panel) and FITC–fibronectin (bottom panel) adhered to the AnoII and HA-modified coupons.

As for albumin, after 15, 120, and 240 min of adhesion, significant differences between the groups can be observed (*p* < 0.05). More specifically, after 15 min, the amounts of albumin are approximately 50% lower in the AnoII coupons compared to the HA-modified coupons. After 30 min of adhesion, this difference decreased to 17%, which was not statistically significant. After 120 min of adhesion, the amounts of protein adhered to the HA-modified samples are approximately 40% higher than those adsorbed onto the AnoII coupons. After 240 min of the coupons’ exposure to FITC–albumin, the HA-modified coupons retained approximately 21% more proteins compared to the controls (*p* < 0.05).

As for fibronectin, after 15 and 30 min of adhesion, the amounts of proteins adhered are approximately 3% and 17% higher for the AnoII coupons compared to the HA-modified coupons, respectively, although no significant difference occurs (*p* > 0.05). Whereas, after 120 min of adhesion, the amount of proteins adhered to the HA-modified coupons was approximately 15% higher than those adhered to the AnoII in the controls, and this was statistically significant (*p* < 0.05). After 240 min, FITC–fibronectin adhered to the HA-modified coupons was approximately 16% higher than that adhered to the AnoII coupons, although this difference is not statistically significant (*p* > 0.05).

Moreover, the washing treatment following 240 min of adherence did not significantly change the amount of FITC protein adhered to both the control and HA-modified samples. Approximately the same amount of proteins have been removed from both HA-modified and AnoII samples.

**Figure 4:**
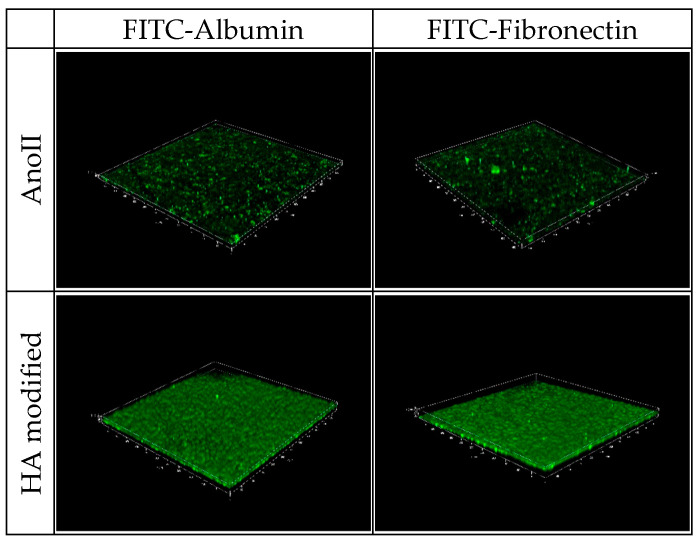
CLSM images of FICT–albumin (**top panel**) and FITC–fibronectin (**bottom panel**) after 240 min of adhesion onto the three Ti6Al4V ELI coupons with anodization type II surface treatment (CTR, control) and Ti6Al4V ELI coupons with anodization type II surface treatment—HA modified (HA modified) (all the replicates performed are reported in Figure S2).

Figure 4 and Figure S1 show the CLSM images of the FITC–albumin and FITC–fibronectin adhered to the two different surfaces after 240 min of contact. Albumin and fibronectin are homogenously distributed onto the HA-modified coupons, while the adhesion of both proteins is localized in a few spots. These qualitative results are in agreement with the quantitative data presented in Figure 3 and confirm that the proteins show a higher affinity to adhere to HA-modified samples. Protein–implant interactions play a crucial part in the early phase of osseointegration, indicating a beneficial impact of the HA surface treatment. This could be related to the hydrophilic properties if the surface combined with the unique needle-shaped surface topography favors the process [23,24].

### 3.3. Proliferation, Adhesion, and Mineralization of Human Osteoblasts and Adipose-Derived Stem Cells

**Figure 5:**
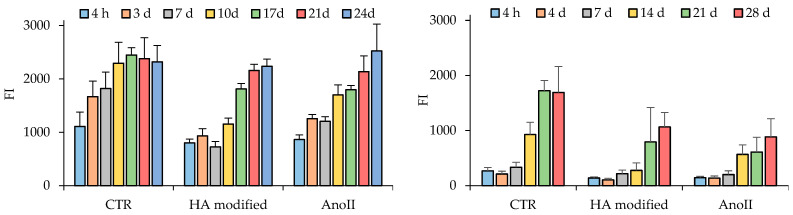
(**Left**) HOB (cell passage 4th) and (**right**) hASC proliferation expressed as fluorescence intensity (FI) of the reduced AlamarBlue™ reagent (mean values, s.d., n = 7) (Statistics are reported in Supplementary Materials).

The HOB and hASC adhesion and proliferation onto the coupons expressed as fluorescence intensities (FI) of the reduced AlamarBlue^TM^ are shown in Figure 5 (Tables S2 and S3—statistical analysis).

**Figure 6:**
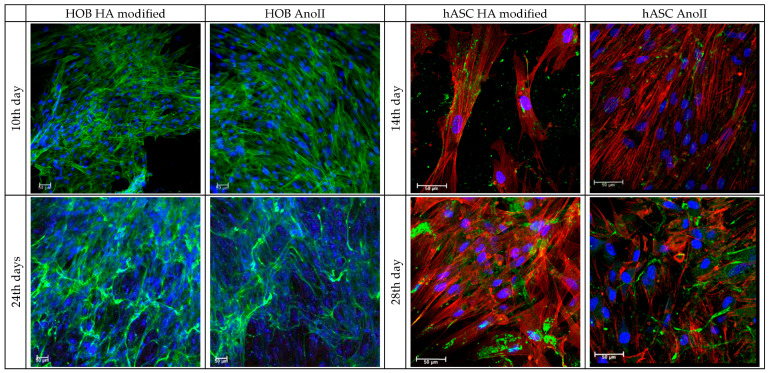
CLSM images of HOBs and hASCs adhered to HA-modified and AnoII coupons. The images were acquired at the beginning and at the end of phase II. Cytoskeletons are green/red and stained with phalloidin, and nuclei are blue with Hoechst.

In all cases, continuous increases in fluorescence intensity can be observed over time, reaching their maximum between 21 and 24–28 days, depending on cell type. At each time point, the HOB population appears to be more numerous in relative comparison to the hACSs. This may be attributed to the bigger cellular dimension of hASCs when compared to HOBs, which limit the number of cells that could occupy the available surface. Apart from the metabolic activity, cell morphology, adhesion, and distribution on the tested surfaces were investigated by CLSM at predefined time points. The results are presented in Figure 6. The adhesion process of cells comprises four distinct steps: attachment, spreading, organization of actin and the cytoskeleton, and finally, the formation of focal adhesion points [25].

After 4 h, despite the higher number of cells in the AnoII samples, the HOBs adhered to both samples are characterized by a rounded shape without a preferred orientation, which is consistent with the very early stage of adhesion [26] (Figure S3). After three days of culture, it is clearly visible that the majority of osteoblasts are elongated, showing a similar behavior in both groups (normal morphology for HOBs) (Figure S3). After 10 days of culture, at the beginning of the differentiation phase, the HOBs start to align, indicating that the surface morphology guides the adhesion and spreading process. The AnoII coupons are characterized by full coverage by osteoblasts, while a few unpopulated spots remain on HA-modified coupons. This observation is in line with the findings of the proliferation assays, indicating fewer cells on the HA-modified samples in relative comparison to the AnoII samples. This behavior may be affected by the solubility of the HA layer shown in Figure 2, possibly affecting the adhesion of HOBs in the in vitro environment. Despite this, HOBs are spatially integrated onto the coupon, showing good adhesion and elongation. After 24 days, the HOBs have completely populated both surfaces. HOBs grown on HA-modified coupons show more distinct elongation and alignment resembling the native shape of the cell.

Similarly, for hASCs, within the early phase of cellular attachment and proliferation (4 h–7 days), cells attached to HA-modified coupons are few and relatively small in size compared to the AnoII group (Figure S4). After 14 days, at the beginning of the differentiation phase, the amount of hASCs on the HA-modified coupons is still less than those identified on the control samples. Yet, following 21 and 28 days, hASCs are visible all over the coupons independently of sample type. Finally, collagen from the ECM can be seen in both groups in similar quantities. Overall, the results suggest that both HA-modified coupons and AnoII coupons feature similar properties in terms of cellular adhesion and proliferation of the cells.

**Figure 7:**
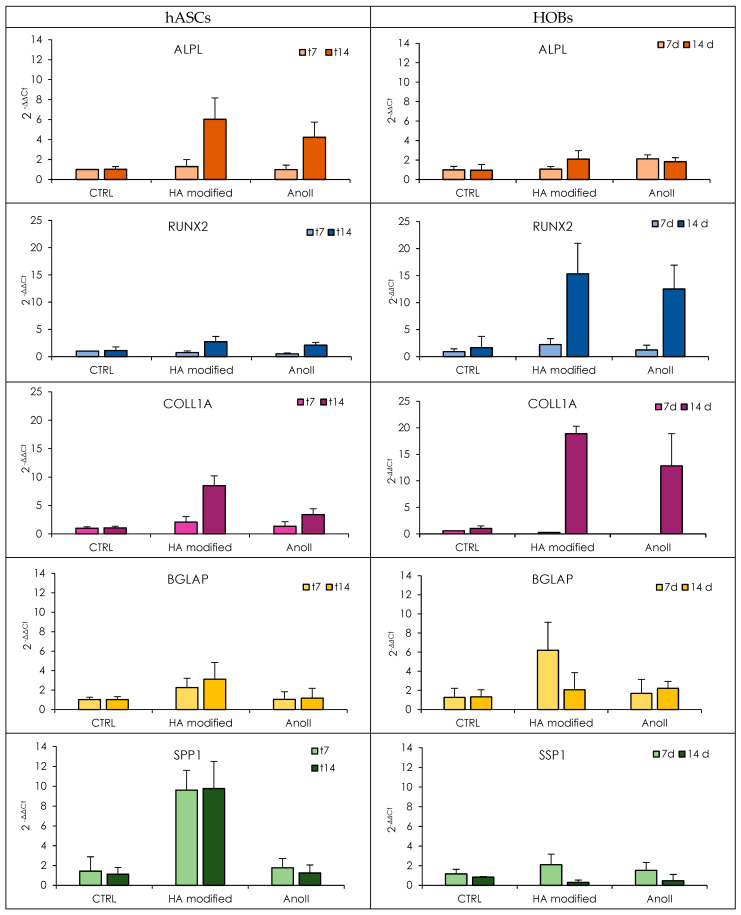
Relative gene expression level (2^−ΔΔCt^) of ALPL, RUNX2, COL1A, BGLAP, and SPP1 at 7 and 14 days from mineralization induction. (Mean values ± s.d., n = 7) HOBs: right panels; hASCs: left panels. Complete statistics are in the Supplementary Materials.

To investigate the differentiation behavior of the cells, the expression of relevant genes was evaluated using rt-qPCR analysis (Figure 7—HOBs left panels and hASCs right panels, Tables S4 and S5—statistical analysis). ECM proteins, in particular, are crucial in mediating cell adhesion to biomaterials [27]. The ECM is a non-cellular 3D structure constructed in the extracellular space and is formed of organic (40%) and inorganic (60%) compounds. The main inorganic component is hydroxyapatite, while the organic ECM consists mainly of collagen I (90%) and non-collagenous proteins (10%). To accommodate for this, the following markers have been considered as part of this investigation: ALPL, in particular, has been selected to investigate the inorganic ECM production, while RUNX2, SSP1, BGLAP, and COL1A have been chosen to evaluate the organic ECM deposition.

The ALPL gene encodes alkaline phosphatase. High expression of ALPL indicates that the mineralization of the extracellular matrix is in process since ALP is a plasma membrane-bound enzyme associated with the increase in the local concentration of phosphate and the inhibition of pyrophosphates; the increase of Ca^2+^ concentration in the presence of ALP determines the hydroxyapatite formation within matrix vesicles and their release in the ECM environment. In terms of ALPL, no differences between the groups are evident after 7 days of differentiation. However, after 14 days, both HOBs and hASCs are characterized by a noticeable increase in ALPL expression, especially when seeded onto HA-modified coupons, although this is less evident in the case of hASCs.

Both HA-modified and AnoII coupons were characterized by a significant increase in the expression of RUNX2 from 7 to 14 days of mineralization. HA-modified samples were characterized by an increased gene expression when compared to AnoII samples for both HOBs and hASCs. Yet, these differences were not found to be statistically significant. However, a remarkable expression of the RUNX2 gene is much more evident in the case of HOBs at 14 days of differentiation. This is a clear indication of cellular differentiation in osteoblasts since the RUNX2 gene is a transcription factor encoding for a nuclear protein with a Runt DNA-binding domain essential for osteoblastic differentiation and skeletal morphogenesis and acts as a scaffold for nucleic acids and regulatory factors involved in skeletal gene expression. RUNX2 plays a key role in the regulation of the osteoblast cell cycle, and higher levels of RUNX2 are present in the G1 phase (the preparatory phase of mitosis).

The COL1A expression of HA-modified samples is significantly higher for both cell types than that of the control. The gene expression reaches its maximum after 14 days of differentiation. Among the monitored genes, COL1A features the highest levels of expression. This appears to be reasonable since collagen is the major organic compound of the ECM and a precursor for the inorganic matrix deposition. This finding is in agreement with the CLSM images (Figure 6), in which collagen was stained green for hASCs.

The second protein identified in high quantities is osteocalcin. This highly abundant bone protein is secreted by osteoblasts and regulates bone remodeling and metabolism. It is encoded by the BGLAP gene. Independently of cell type, BGLAP expression is higher when exposed to HA-modified coupons. It is conceivable that the HA layer promotes the synthesis of Gla (gamma carboxyglutamate) domains, which bind to calcium and hydroxyapatite.

Along with BGLAP, the SPP1 gene is involved in the expression of proteins that have a high affinity for binding to hydroxyapatite. The SPP1 gene encodes osteopontin. The osteopontin–sialoprotein complex regulates the mineralization process in bone. Both cell types grown onto HA-modified coupons are characterized by high SPP1 expression; nevertheless, this is less evident for HOBs. SSP1 gene expression reaches a maximum at the 7th day of differentiation for HOBs, while hASCs show high SPP1 gene expression throughout the experiment.

In summary, the presence of a nanocrystalline hydroxyapatite layer promoted the expression of both genes related to the deposition of inorganic and organic ECM. Similar results have been reported by Knabe et al., which demonstrated that an ultra-thin hydroxyapatite surface layer enhances osteogenesis by inducing the highest expression of osteogenic markers [28]. The effect is remarkable in the case of hASCs. For this reason, SEM analysis was performed on the 14th day to investigate how the adhesion and grown substrate influence the cell anchorage [29].

**Figure 8:**
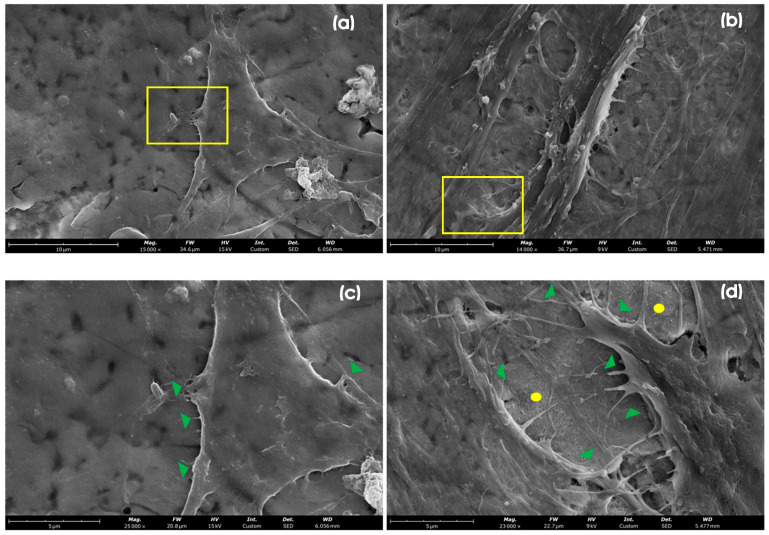
SEM images of hASCs adhered and grown onto Ano II ((**a**,**c**): **left**) and HA-modified ((**b**,**d**): **right**) coupons at low ((**a**,**b**): **top panels**) and high ((**c**,**d**): **bottom panels**) magnifications on the 14th day of differentiation (green arrows: filipodia; yellow circle: nano hydroxyapatite background).

Figure 8 shows the results of the SEM investigation where hASCs have adhered and grown onto Ano II and HA-modified coupons on the 14th day of differentiation. The hASCs do not show confluency and, consequently, direct contact with the surrounding cells. A few short filipodia (marked with green triangles, Figure 8b) are present at the cell border to establish contact with the AnoII coupon. Considering this, despite high cell proliferation and high expression of genes encoding ECM proteins, the cells remain isolated. On the contrary, hASCs attached to the HA-modified coupon are characterized by an impressive amount of filipodia (green arrows, Figure 8d) having a thick structure and a long extension all over the coupon surface. Even the HA nanocrystals can be noticed in the background (yellow circle, Figure 8d). The specific nanotopography is most certainly the major contributor for the differences in cell anchoring and spreading across the surface. Conversely to what is evidenced in the HA dissolution, the presence of cells seems to create a microenvironment onto the HA layer and to slow down its dissolution. This finding and the higher expression of genes encoding ECM proteins suggest that the HA layer effectively enhances osteodifferentiation and osteointegration [30,31].

## 4. Conclusions

Ti6Al4V ELI coupons treated with an ultra-thin layer of nanocrystalline HA coating support the anchorage, attachment, spreading, and proliferation of both human osteoblasts (HOBs) and human adipose stem cells (hASCs). The efficacy of the hydroxyapatite coating could be influenced by the initial surface roughness of the titanium alloy; for example, excessively rough surfaces could hinder and impair a uniform distribution of the HA coating, potentially affecting its adhesion and long-term stability. Qualitative imaging of the test samples confirms a high number of cells and suggests a normal organization of the cytoskeleton. Overall, the behavior of the cells was found to be similar between both test samples (HA modified) and the controls (AnoII). Yet, coupons treated with nanocrystalline HA were associated with enhanced gene expression relevant for the synthesis of the inorganic and organic components of the ECM and bone when compared to their unmodified counterparts. In particular, hASCs were able to promote ECM production, leading to a higher expression of collagen I and non-collagenous proteins, which is crucial to regulating mineral matrix formation. Moreover, they present an impressive amount of filipodia, having a long extension all over the coupon surface. Ultimately, these findings suggest that the HA treatment effectively enhances the osteoconductive properties of implant materials through improved osseous differentiation and cellular maturation.

## Data Availability

All data generated or analyzed during this study are included in this published article and its Supplementary Information files. Stryker Content ID is OT-AR-31, 07-2024.

## Supplementary Materials

The following supporting information can be downloaded at: https://www.mdpi.com/article/10.3390/nano14141181/s1. Figure S1: EDS spectra of the Ti6Al4V ELI coupons (with anodization type II surface treatment, and with anodization type II surface treatment—HA modified). Figure S2: CLSM images of FICT-albumin (top panel) and FITC-fibronectin (bottom panel) after 240 min of adhesion onto the three Ti6Al4V ELI coupons with anodization type II surface treatment (CTR, control) and Ti6Al4V ELI coupon with anodization type II surface treatment—HA modified (HA mod). Figure S3: CLSM images of HOB adhered on the different test and control coupons. The images were acquired after 4 hours, 3 days, 10 days, 10 days, and 24 days of culture. Cytoskeleton in green stained with FITC and nuclei in blue with Hoechst. Figure S4: CLSM images of HOB adhered on the different test and control coupons. The images were acquired after 4 hours, 4 days, 7, days, 10 days, 14 days, 21 days, and 28 days of culture. Cytoskeleton in green stained with FITC and nuclei in blue with Hoechst. Table S1: List of the primers and relative gene sequence description. Table S2. Statistical analysis of HOB proliferation. Table S3. Statistical analysis of ADSCs proliferation. Table S4. Statistical analysis of relative gene expression level (2^−ΔΔCt^) of RUNX2 (a), ALPL (b), SPP1 (c), BGLAP (d), COL1A (e) at 7 and 14 days from mineralization induction; p* values of CTRL vs Hap. Table S5. Statistical analysis of relative gene expression level (2^−ΔΔCt^) of RUNX2 (a), ALPL (b), SPP1 (c), BGLAP (d), COL1A (e) at 7 and 14 days from mineralization induction.
