# Supplementary material for: Hydroxyapatite-Coated Ti6Al4V ELI Alloy: In Vitro Cell Adhesion
Source: Nanomaterials (Basel). 2024 Jul 11;14(14):1181. doi: 10.3390/nano14141181 (PMC11279432; doi:10.3390/nano14141181)
Supplement: Supplementary file 1 [file nanomaterials-14-01181-s001.zip › nanomaterials-3033190-supplementary.pdf]

Hydroxyapatite-coated Ti6Al4V ELI alloy: in vitro cell adhesion

Marco Ruggeri[1,§], Dalila Miele[1,§], Laura Caliogna[2], Eleonora Bianchi[1], Johannes Maui Jepsen[3], Barbara Vigani[1], Silvia Rossi[1], Giuseppina Sandri[1,*]

[1] Department of Drug Sciences, University of Pavia, Viale Taramelli 12, 27100 Pavia, Italy

[2] Orthopedy, Fondazione IRCCS Policlinico San Matteo, 27100 Pavia, Italy

[3] Stryker Trauma GmbH, Professor Küntscher-Straße 1-5, 24232, Schönkirchen, Germany

§: equally contributed

* Correspondence: g.sandri@unipv.it

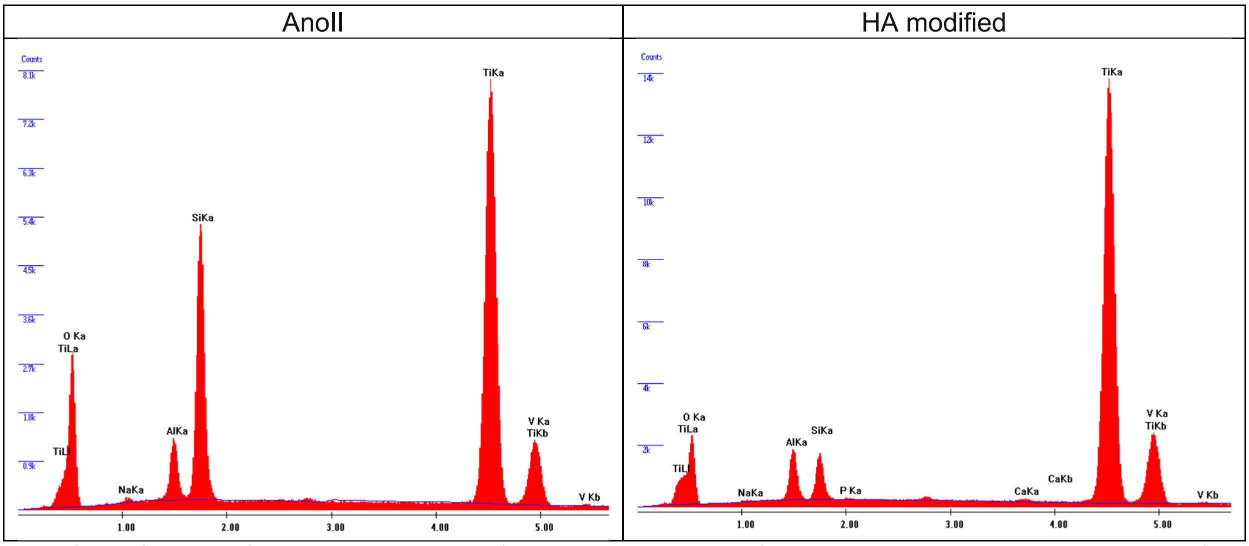

Figure S1: EDS spectra of the Ti6Al4V ELI coupons (with anodization type II surface treatment, and with anodization type II surface treatment - HA modified).

Table S1: List of the primers and relative gene sequence description.

Primer	Abbreviation	Amplicon Context Sequence	Amplicon Length (bp)
Runt-related transcription factor 2	RUNX2	AAGTAGCTACCTATCACAGAGCAATTAAAGTTACAGTAGA<br>TGGACCTCGGGAACCCAGAAGGCACAGACAGAAGCTTGA<br>TGACTCTAAACCTAGTTTGTTCTCTGACCGC	80
Alkaline phosphatase	ALPL	CATCGGGCAGGCAGGCAGCTTGACCTCCTCGGAAGACAC<br>TCTGACCGTGGTCACTGCGGACCATTCCCACGTCTTCACA<br>TTTGGTGGATAC	61
Secreted phosphoprotein 1	SPP1	GTTTCGCAGACCTGACATCCAGTACCCTGATGCTACAGAC<br>GAGGACATCACCTCACACATGGAAAGCGAGGAGTTGAAT<br>GGTGCATACAAGGCCAT	66
Bone gamma-carboxyglutamate (gla) protein	BGLAP	GCGGGGCAGGCCAGCTGAGTCCTGAGCAGCAGCCCAGC<br>GCAGCCACCGAGAC<br>ACCATGAGAGCCCTCACACTCCTCGCCCTATTGGCCCTG<br>GCCGCACT	69
Collagen, type I, alpha 1	COL1A1	TCTTGGTCTCGTCACAGATCACGTCATCGCACAACACCTT<br>GCCGTTGTCGCAGACGCAGATCCGGCAGGGCTCGGGTTT	113

		CCACACGTCTCGGTCATGGTACCTGAGGCCGTTCTGTAC GCAGGTGATTGGTGGGATGTCTTCG	
Glyceraldehyde-3-phosphate dehydrogenase	GAPDH	GTATGACAACGAATTTGGCTACAGCAACAGGGTGGTGGA CCTCATGGCCCACATGGCCTCCAAGGAGTAAGACCCCTG GACCACCAGCCCCAGCAAGAGCACAAGAGGAAGAGAGA GACCCTCACTGCTGGGGAGTCCCTGCCACAC	117

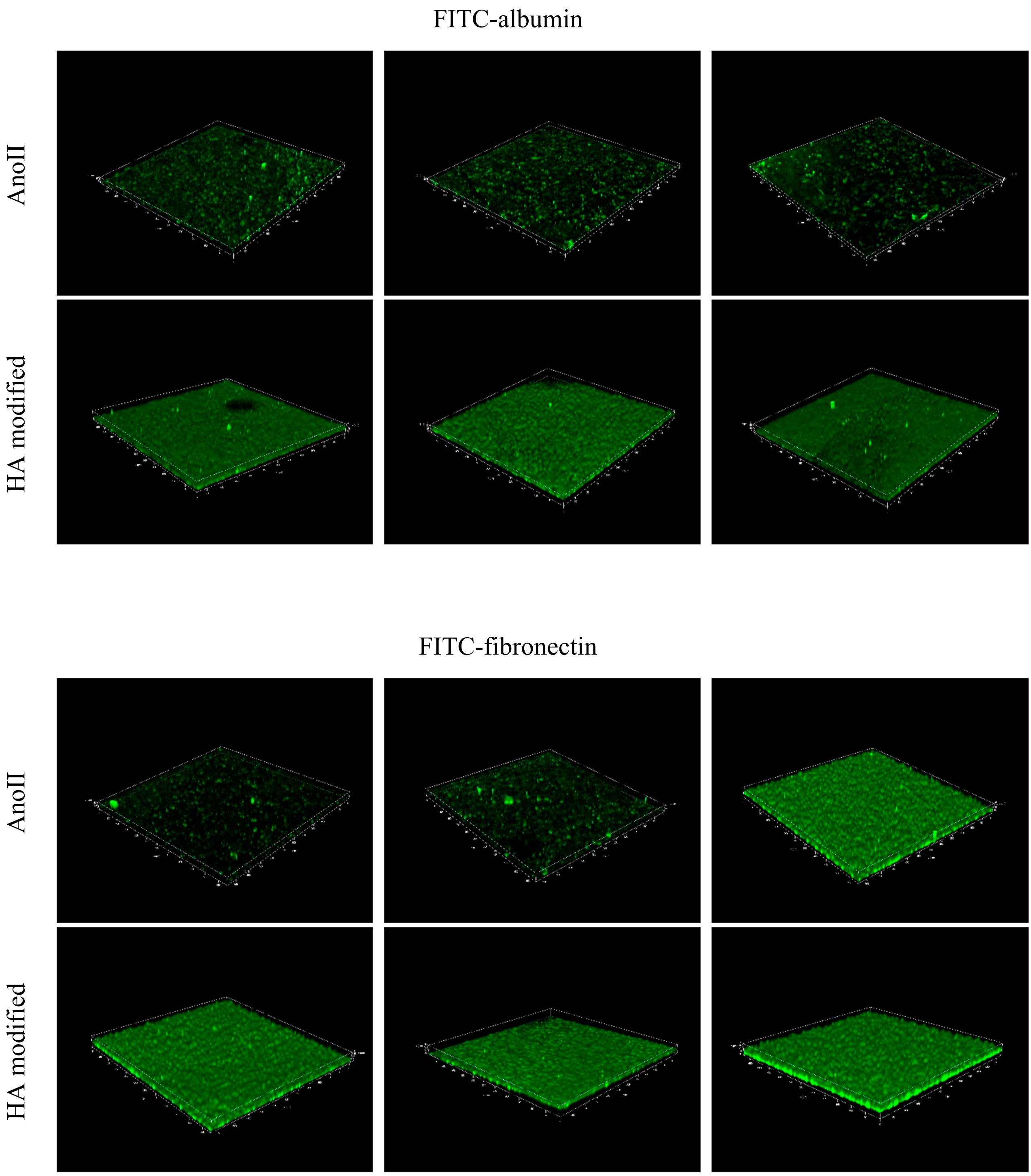

Figure S2: CLSM images of FICT-albumin (top panel) and FITC-fibronectin (bottom panel) after 240 min of adhesion onto the three Ti6Al4V ELI coupons with anodization type II surface treatment (CTR, control) and Ti6Al4V ELI coupon with anodization type II surface treatment - HA modified (HA mod).

Table S2. Statistical analysis of HOB proliferation.

	4h	3d	7d	10d	17d	24d
CTRL vs HAp	<0.05	<0.05	<0.05	<0.05	<0.05	<0.05
CTRL vs AnoII	<0.05	<0.05	<0.05	<0.05	<0.05	<0.05
HAp vs AnoII	insignificant	<0.05	<0.05	<0.05	insignificant	insignificant

Table S3. Statistical analysis of ADSCs proliferation.

	4h	3d	7d	10d	17d	24d
CTRL vs HAp	<0.05	<0.05	<0.05	<0.05	<0.05	<0.05
CTRL vs AnoII	<0.05	<0.05	<0.05	<0.05	<0.05	<0.05
HAp vs AnoII	insignificant	<0.05	insignificant	<0.05	<0.05	insignificant

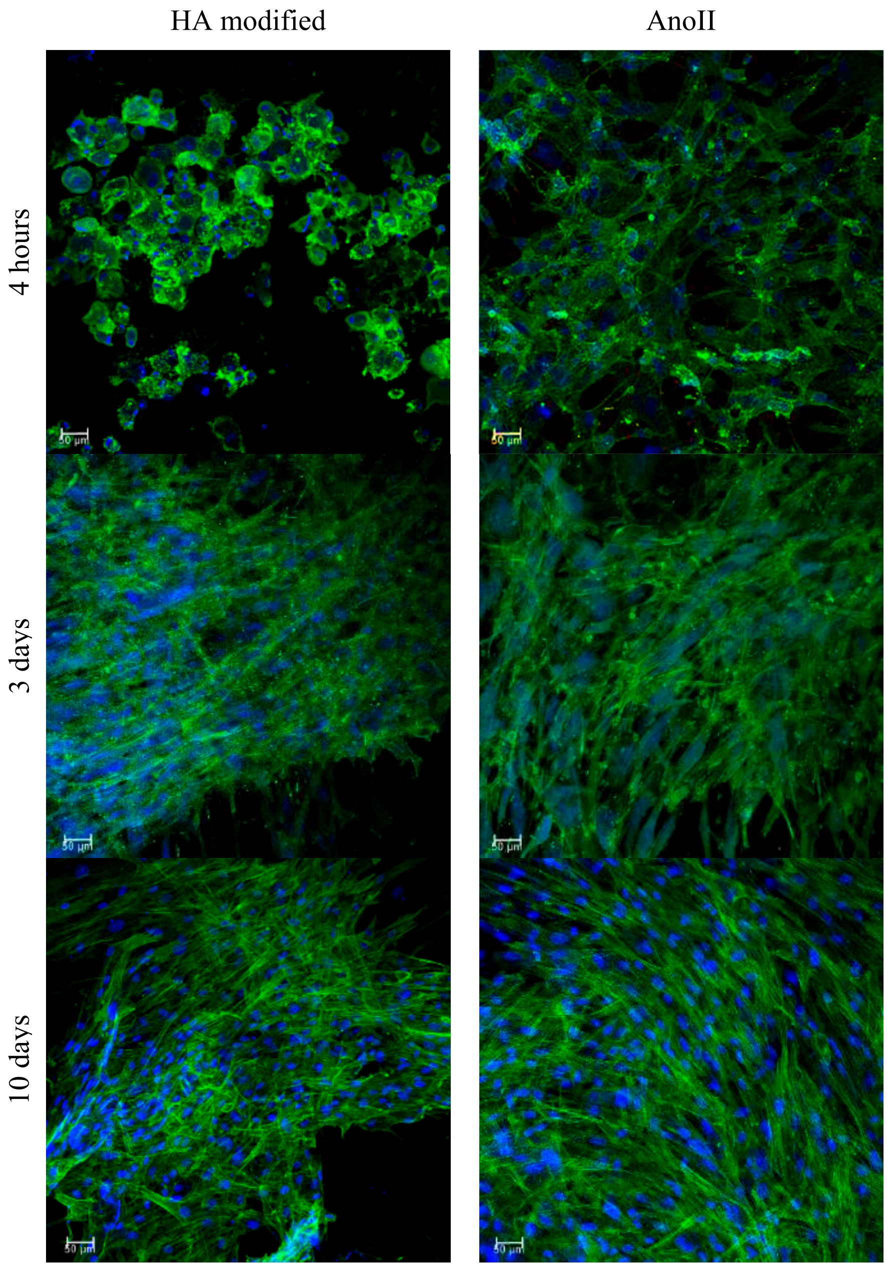

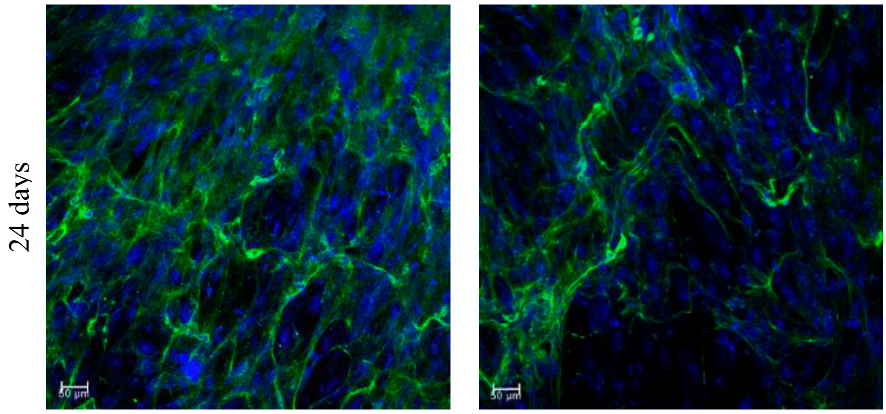

Figure S3: CLSM images of HOB adhered on the different test and control coupons. The images were acquired after 4 hours, 3 days, 10 days, 10 days, and 24 days of culture. Cytoskeleton in green stained with FITC and nuclei in blue with Hoechst.

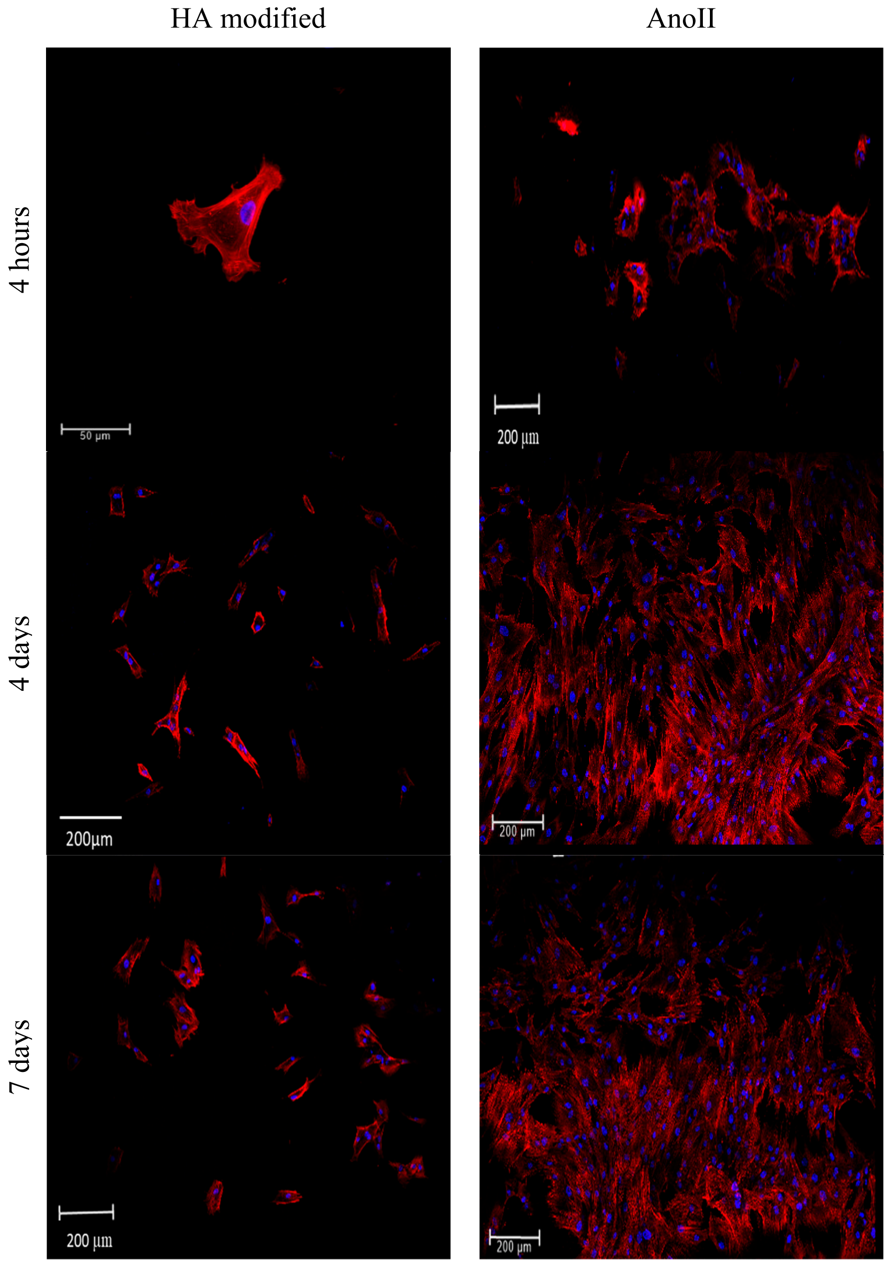

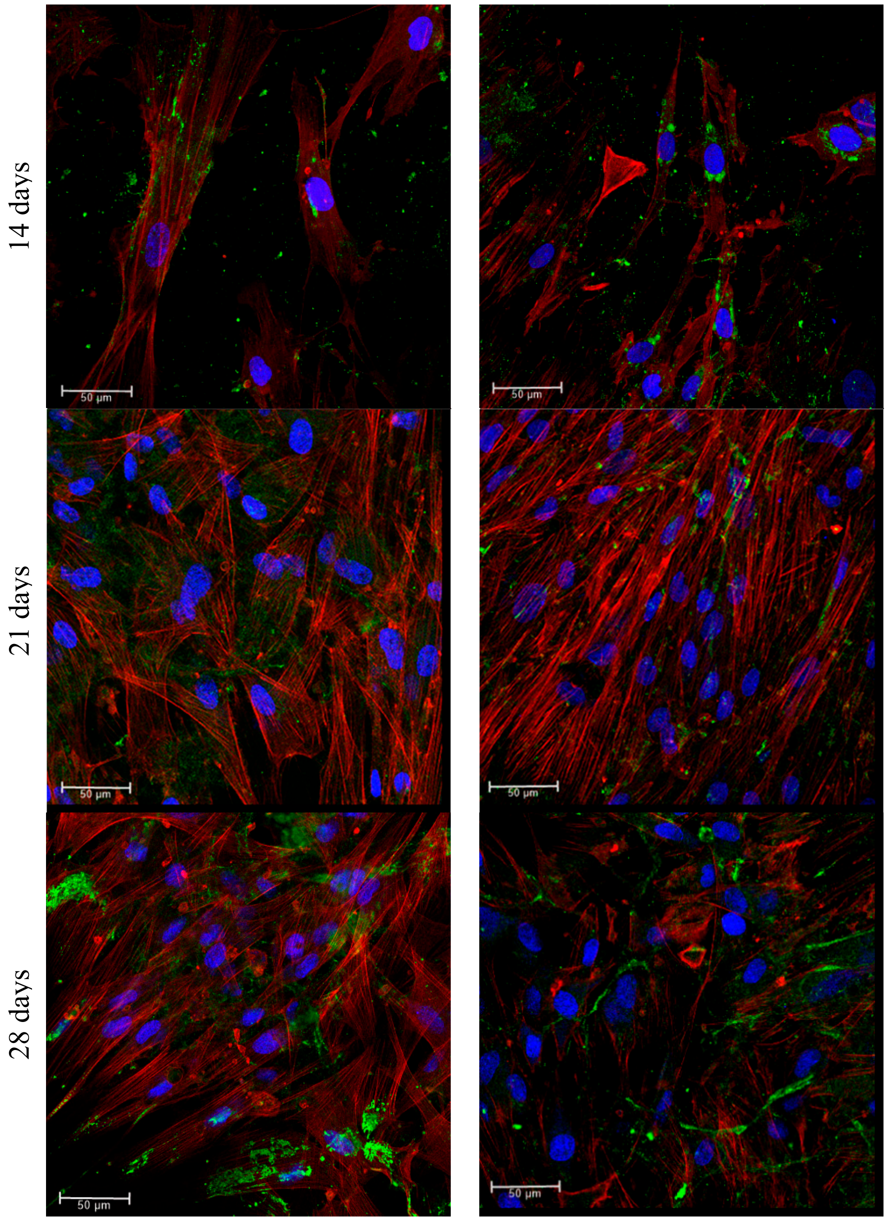

Figure S4: CLSM images of HOB adhered on the different test and control coupons. The images were acquired after 4 hours, 4 days, 7, days, 10 days, 14 days, 21 days, and 28 days of culture. Cytoskeleton in green stained with FITC and nuclei in blue with Hoechst.

Table S4. Statistical analysis of relative gene expression level (2-ΔΔCt) of RUNX2 (a), ALPL (b), SPP1 (c), BGLAP (d), COL1A (e) at 7 and 14 days from mineralization induction; p* values of CTRL vs HAp

7 days	RUNX 2	ALPL	SPP1	BGLAP	COL1A
CTRL vs HAp	insignificant	insignificant	<0.05	insignificant	insignificant
CTRL vs AnoII	insignificant	insignificant	insignificant	insignificant	insignificant
HAp vs AnoII	insignificant	insignificant	<0.05	insignificant	insignificant
14 days	RUNX 2	ALPL	SPP1	BGLAP	COL1A
CTRL vs HAp	insignificant	<0.05	<0.05	insignificant	<0.05
CTRL vs AnoII	insignificant	<0.05	insignificant	insignificant	insignificant
HAp vs AnoII	insignificant	insignificant	<0.05	<0.05	<0.05

Table S5. Statistical analysis of relative gene expression level (2-ΔΔCt) of RUNX2 (a), ALPL (b), SPP1 (c), BGLAP (d), COL1A (e) at 7 and 14 days from mineralization induction.

7 days	RUNX 2	ALPL	SPP1	BGLAP	COL1A
CTRL vs HAp	insignificant	insignificant	insignificant	<0.05	insignificant
CTRL vs AnoII	insignificant	<0.05	insignificant	insignificant	insignificant
HAp vs AnoII	insignificant	<0.05	insignificant	<0.05	insignificant
14 days	RUNX 2	ALPL	SPP1	BGLAP	COL1A
CTRL vs HAp	<0.05	<0.05	<0.05	<0.05	<0.05
CTRL vs AnoII	<0.05	<0.05	<0.05	<0.05	<0.05
HAp vs AnoII	<0.05	<0.05	<0.05	<0.05	<0.05
